# PGE**_2_** Desensitizes ****β****-Agonist Effect on Human Lung Fibroblast-Mediated Collagen Gel Contraction through Upregulating PDE4

**DOI:** 10.1155/2013/145197

**Published:** 2013-10-08

**Authors:** Qiuhong Fang, Yingmin Ma, Jing Wang, Joel Michalski, Stephen I. Rennard, Xiangde Liu

**Affiliations:** ^1^Department of Pulmonary and Critical Care, Beijing Shijitan Hospital, Capital Medical University, Beijing 100038, China; ^2^Pulmonary, Critical Care, Sleep and Allergy Division, Department of Internal Medicine, University of Nebraska Medical Center, Omaha, NE 68198-5910, USA

## Abstract

In the current study, we investigated the effect of a long-acting **β**-agonist (salmeterol) and a phosphodiesterase 4 (PDE4) inhibitor (cilomilast) on human lung fibroblast-mediated collagen gel contraction. Higher concentrations of salmeterol (10^−7^ and 10^−6^ M) inhibited fibroblast-mediated collagen gel contraction. No effect was observed with cilomilast alone (up to 10^−5^ M). In the presence of 10^−8^ M salmeterol, however, cilomilast could significantly inhibit fibroblast-mediated collagen gel contraction in a concentration-dependent manner (10^−7^ *~*10^−5^ M). Blockade of endogenous PGE_2_ by indomethacin further potentiated the inhibitory effect of salmeterol on fibroblast-mediated collagen gel contraction, but it did not affect cilomilast's effect. Pretreatment with PGE_2_ abolished the inhibitory effect of salmeterol, but it potentiated the inhibitory effect of cilomilast on fibroblast-mediated collagen gel contraction. Finally, indomethacin slightly inhibited PDE4C expression, while PGE_2_ stimulated the expression of PDE4A and -4C in human lung fibroblasts. These findings suggest that long-acting **β**-agonist and PDE4 inhibitor have a synergistic effect in regulating fibroblast tissue repair functions and that PGE_2_ can modulate the effect of **β**-agonist and PDE4 inhibitor at least in part through the mechanism of regulating PDE4 expression.

## 1. Introduction

Lung fibroblasts play an important role in the repair processes following lung injury and contribute to the pathogenesis of chronic obstructive pulmonary disease (COPD). Impaired repair function of lung fibroblasts could result in emphysema, while overproduction of extracellular matrix by fibroblasts may lead to peribronchial fibrosis, both of which are major pathologic characteristics of COPD [1, 2]. Fibroblast-mediated collagen gel contraction is an *in vitro* model of tissue remodeling and is widely used to investigate repair functions of fibroblasts from a variety of tissues including lung and skin [3, 4].

Fibroblast-mediated collagen gel contraction can be modulated by many factors. For example, TGF-*β*1 and PDGF are potent stimulators of fibroblast-mediated collagen gel contraction, while prostaglandin E_2_ (PGE_2_) and *β*-adrenergic agonists are inhibitors of this process [5–8]. The inhibitory effect of *β*-agonist exposure on fibroblast-mediated collagen gel contraction is based on increased intracellular cAMP levels [7]. Lung fibroblasts are also important sources of PGE_2_, which modulates the level of intracellular cAMP levels and thus modulates repair functions through autocrine and paracrine pathways [2, 9].

The intracellular level of cAMP is also dependent on its rate of degradation. Phosphodiesterases (PDEs) are enzymes that degrade cAMP through hydrolysis. PDEs comprise 11 related gene families that have been classified according to their amino acid sequences and substrate specificities. Among the PDE families, the PDE4 family, which includes four isoforms named PDE4A, -4B, -4C, and -4D, specifically hydrolyzes cAMP [10, 11]. Several studies have shown that upregulation of PDE4 activity reduces cell sensitivity to the cAMP-elevating reagents such as *β*-agonist [10, 12–14]. 

We have shown that PGE_2_ exposure augments PDE4 activity and induces functional homologous and heterologous attenuation of cAMP-mediated inhibition of fibroblast chemotaxis [15]. Similarly, PDE4 activity induced by PGE_2_ is involved in the desensitization of *β*-mimetics in human myometrium during the late stages of pregnancy [16]. Moreover, salbutamol also upregulates PDE4 activity and induces a heterologous desensitization of U937 cells to PGE_2_ [14]. Thus, we hypothesize that endogenous and exogenous PGE_2_ may reduce sensitivity of the lung fibroblasts to long-acting *β*-agonist through upregulating PDE4.

We have previously reported that *β*-adrenergic agonists and PDE4 inhibitors can modulate fibroblast-mediated collagen gel contraction through the cAMP/PKA pathway [7, 17]. The synergistic effect of *β*-agonist and PDE4 inhibitor on fibroblast-mediated collagen gel contraction and its mechanism, however, has not been investigated. In the current paper, we first studied the effect of varying concentrations of salmeterol plus cilomilast or vice versa on lung fibroblast-mediated collagen gel contraction. Secondly, we studied the role of endogenous and exogenous PGE_2_ in regulating the synergistic effect of salmeterol and cilomilast on fibroblast-mediated collagen gel contraction.

## 2. Materials and Methods

### 2.1. Materials

Salmeterol and cilomilast were kindly provided by GlaxoSmithKline (Upper Marrion, PA, USA). Indomethacin, PGE_2_, and anti-*β*-actin antibody were purchased from Sigma (St. Louise, MO, USA). Anti-PDE4 antibodies were purchased from Abcam (PDE4A: cat#: ab14607; PDE4B: cat#: ab14611; PDE4C: cat#: ab97356; PDE4D: cat#: ab14613, Abcam, Inc., Cambridge, MA, USA). Native type I collagen, rat tail tendon collagen (RTTC), was extracted from rat tail tendon as described previously [7].

### 2.2. Cell Culture

Human fetal lung fibroblast (HFL-1) was purchased from the American Type Culture Collection (ATCC, Rockville, MD, USA). Cells were cultured in DMEM supplemented with 10% FCS, penicillin/streptomycin, and fungizone. Passages of 14 through 20 were used for the experiments.

### 2.3. Collagen Gel Preparation and Contraction Assay

Gels were prepared using a previously published method [18]. Briefly, distilled water, 4X concentrated DMEM and RTTC were mixed so that the final mixture resulted in a physiological ionic strength, that is, 1X DMEM, and a pH of 7.4. Cells were suspended with serum-free DMEM (SF-DMEM) at a density of 10^7^ cells/mL and then mixed with the RTTC solution mentioned above so that the final cell density in the collagen solution was 4 × 10^5^ cells/mL and the final concentration of collagen was 0.75 mg/mL. For pretreatment with PGE_2_ or indomethacin, fibroblast monolayers were refed with SF DMEM supplemented with or without PGE_2_ or indomethacin and then incubated for 24 hours prior to harvest. Aliquots of the mixture of fibroblasts and collagen were then cast into each well of a 24-well tissue culture plate (0.5 mL/well). After 20 minutes for gelation at room temperature, the gels were released into 60 mm tissue culture dishes (three gels per each dish), which contained 5 mL of freshly prepared SF-DMEM with or without salmeterol or cilomilast as indicated. The gels were then incubated at 37°C in a 5% CO_2_ atmosphere for 2–5 days as indicated, and the area of each gel was measured with an Optomax V Image Analyzer (Optomax, Burlington, MA, USA) daily. Data were expressed as the percentage of the size compared to original gel size.

### 2.4. Immunoblotting

Immunoblotting for PDE4A, -4B, -4C, -4D and for *β*-actin was carried out with whole cell lysates obtained from the cells pretreated with either PGE_2_ or indomethacin for 24 hrs. Briefly, cells were plated into 60 mm dishes and cultured till confluent. Cells were then treated with SF-DMEM supplemented with ethanol (1 : 1000 dilution as reagent dissolvent), PGE_2_ (10^−8^ M), or indomethacin (10^−6^ M). After 24 hrs of culturing, cells were washed once with cold PBS and then treated with cell lysis buffer (35 mM Tris. HCl, pH 7.4, 0.4 mM EGTA, 10 mM MgCl_2_, and 1 : 1000 of proteinase inhibitor cocktail). Lysates were briefly sonicated on ice and centrifuged at 10,000 g for 5 min at 4°C. PAGE-SDS (10%) gels were prepared, and 10 *μ*g/lane of total protein was loaded. The resolved proteins were transferred to PVDF membrane and incubated with primary antibodies at 1 : 500 dilution. After incubation with HRP-conjugated 2nd antibodies, images were visualized with Lumigen PS-3 (Beckman, Southfield, MI, USA) and quantified with a Typhon Scanner (Amersham Pharmacia Biotech, Little Chalfont, Buckinghamshire, England, UK). 

### 2.5. Statistical Analysis

All experiments were performed on three separate occasions, each of which included triplicate gels for each condition. All data are expressed as mean ± standard error of the mean. Statistical comparisons of multi-group data were analyzed by analysis of variance (ANOVA) followed by Bonferroni's (two-way) posttest correction using PRISM4 software. 

## 3. Result

**Figure 1:**
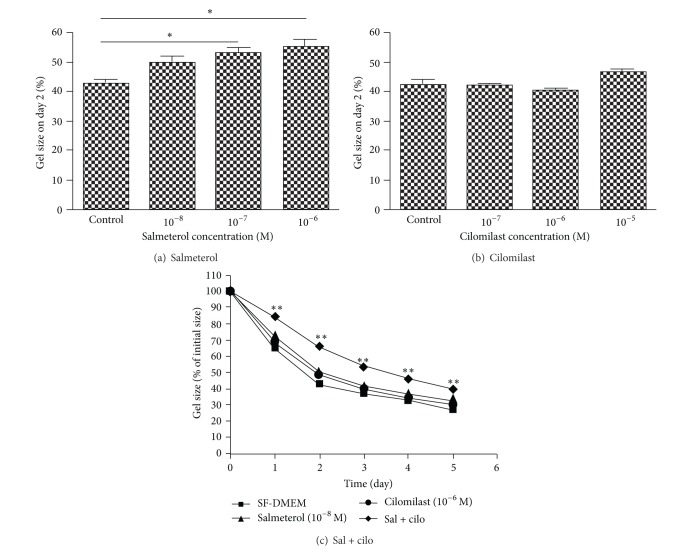
Synergistic effect of salmeterol and cilomilast on lung fibroblast-mediated collagen gel contraction. HFL-1 cells were cast into collagen gels and released into SF-DMEM containing either varying concentrations of salmeterol (0, 10^−8^, 10^−7^ and 10^−6^ M, (a)) or cilomilast (0, 10^−7^, 10^−6^, and 10^−5^ M, (b)) as indicated. Gel size was measured on day 2. Vertical axes: gel size was expressed as percentage of initial size on day 2 (%); horizontal axes: concentrations of salmeterol or cilomilast. *P < 0.05. (c) Synergistic effect of salmeterol and cilomilast are shown. HFL-1 cells were cast into collagen gels and released into SF-DMEM containing salmeterol (10^−8^ M) and/or cilomilast (10^−6^ M). Gel size was measured daily for 5 days. Vertical axis: gel size was expressed as percentage of initial size (%); horizontal axis: time (day). **P < 0.01 compared to SF-DMEM at each time point.

Human lung fibroblast- (HFL-1-) mediated collagen gel contraction, an *in vitro* model of tissue remodeling, was used to investigate the effect of a cAMP-elevating agent on fibroblast tissue repair functions. The long-acting *β*-agonist salmeterol inhibited fibroblast-mediated collagen gel contraction in a concentration-dependent manner (10^−8^ ~ 10^−6^ M; *P* < 0.05 at concentrations of 10^−7^ and 10^−6^ M compared to control, Figure 1(a)). By contrast, a PDE4 inhibitor, cilomilast, had no effect on fibroblast-mediated collagen gel contraction when it was added alone at concentrations of 10^−7^, 10^−6^, and 10^−5^ M (Figure 1(b)). When salmeterol (10^−8^ M) and cilomilast (10^−6^ M), concentrations that were without effect when added alone, were added together, fibroblast-mediated collagen gel contraction was significantly inhibited from day 1 through day 5 (*P* < 0.01, Figure 1(c)).

**Figure 2:**
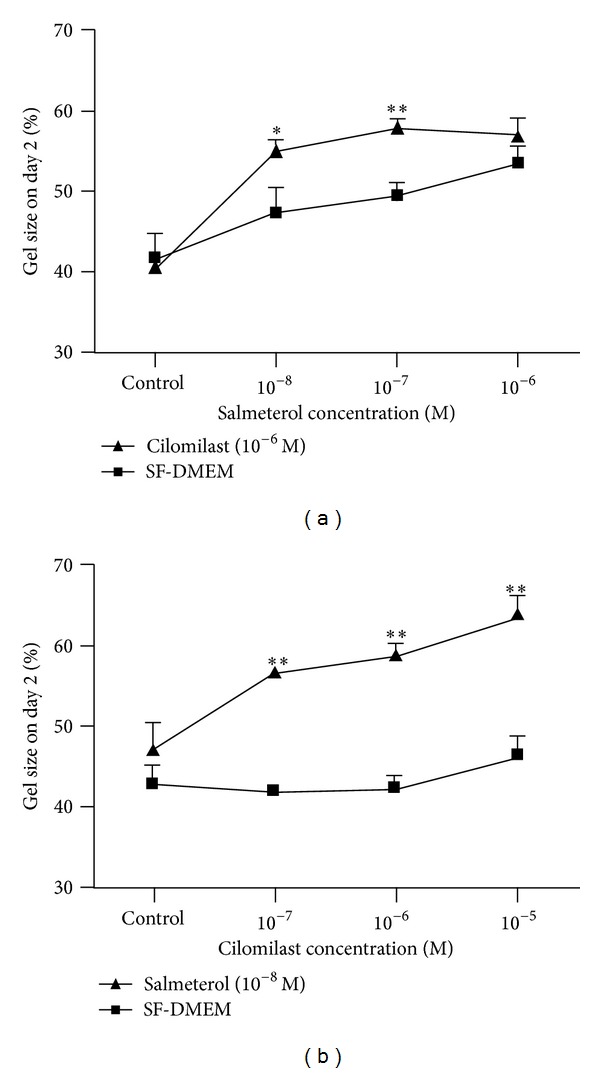
Cilomilast potentiates the inhibitory effect of salmeterol on fibroblast-mediated collagen gel contraction. (a) Effect of cilomilast on varying concentrations of salmeterol. HFL-1 cells were cast into collagen gels and released into SF-DMEM containing varying concentrations (0, 10^−8^, 10^−7^, and 10^−6^ M) of salmeterol in the presence or absence of cilomilast (10^−6^ M). Vertical axis: gel size on day 2 (%); horizontal axis: salmeterol concentration (M). Square: SF-DMEM only; triangle: in the presence of 10^−6^ M cilomilast. *P < 0.05; **P < 0.01 compared to salmeterol only. (b) Effect of salmeterol in the presence of varying concentrations of cilomilast. HFL-1 cells containing collagen gels were released into SF-DMEM supplemented with 10^−8^ M salmeterol in the presence or absence of varying concentrations (0, 10^−7^, 10^−6^, and 10^−5^ M) of cilomilast. Vertical axis: gel size on day 2 (%); horizontal axis: cilomilast concentrations (M). Square: SF-DMEM only; triangle: in the presence of salmeterol (10^−6^ M). **P < 0.01 compared to cilomilast alone at each time point.

Next, we examined the combination of varying concentrations of salmeterol plus one concentration of cilomilast, or vice versa, and the resultant effect on collagen gel contraction. In the presence of cilomilast (10^−6^ M), lower concentrations of salmeterol (10^−8^ and 10^−7^ M) significantly (10^−8^ M, *P* < 0.05; 10^−7^ M, *P* < 0.01) inhibited fibroblast-mediated collagen gel contraction compared to salmeterol alone (Figure 2(a)). While cilomilast alone at concentrations of 10^−7^ ~ 10^−5^ M had no effect on fibroblast-mediated collagen gel contraction, in the presence of a low concentration of salmeterol (10^−8^ M) cilomilast at all three concentrations tested (10^−7^, 10^−6^, and 10^−5^ M) significantly inhibited collagen gel contraction (*P* < 0.01, Figure 2(b)).

**Figure 3:**
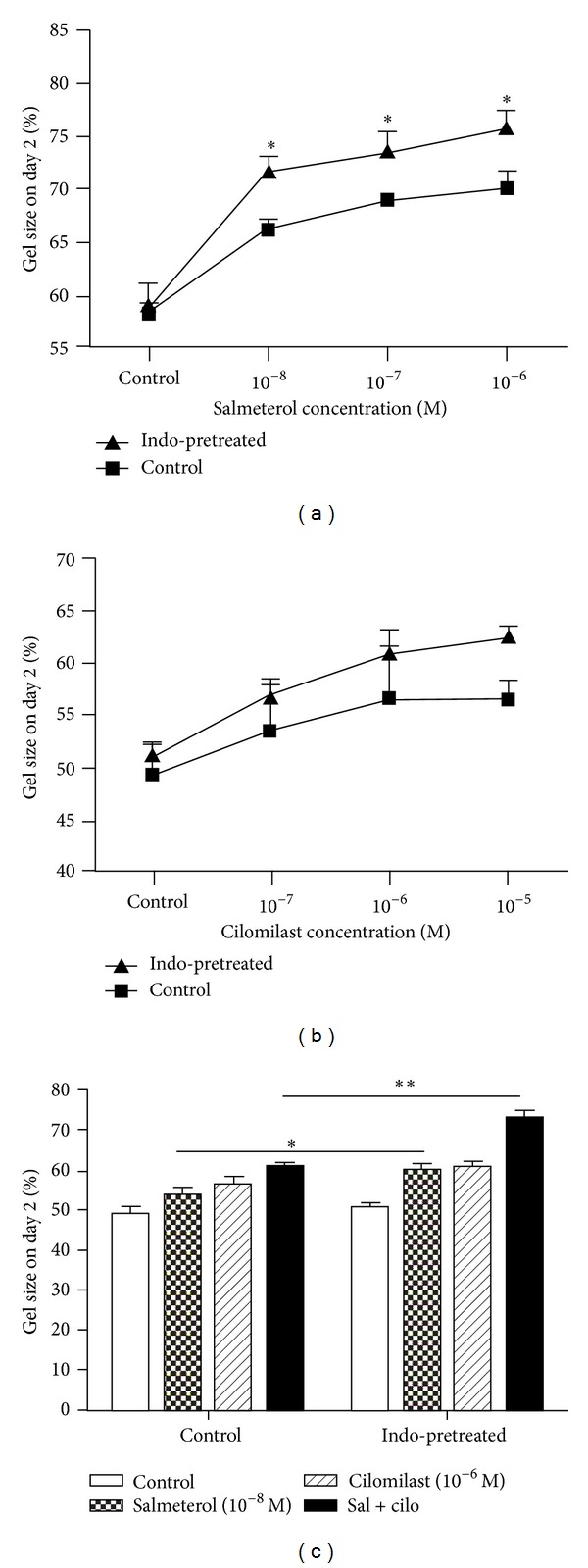
Indomethacin potentiates salmeterol but not cilomilast. Confluent HFL-1 cells were pretreated with SF-DMEM with or without 10^−6^ M indomethacin for 24 hrs. The next day, cells were cast into collagen gels as described in Section 2. (a) Indomethacin pretreatment potentiated the salmeterol effect on fibroblast-mediated collagen gel contraction. Gels were released into SF-DMEM supplemented with varying concentrations of salmeterol as indicated. Vertical axis: gel size on day 2 (%); horizontal axis: salmeterol concentrations (M). Square: cells were cultured in SF-DMEM for 24 hrs prior to being cast into collagen gels; triangle: cells were pretreated with 10^−6^ M indomethacin for 24 hrs prior to being cast into collagen gels. *P < 0.05 compared to control at each time point. (b) Indomethacin pretreatment did not affect the cilomilast effect. Gels were released into SF-DMEM containing varying concentrations of cilomilast as indicated. Vertical axis: gel size on day 2 (%); horizontal axis: cilomilast concentrations (M). Square: cells were cultured in SF-DMEM for 24 hrs prior to being cast into collagen gels; triangle: cells were pretreated with 1 *μ*M indomethacin for 24 hrs prior to being cast into collagen gels. (c) Indomethacin further potentiated the synergistic effect of salmeterol and cilomilast added together on fibroblast-mediated collagen gel contraction. Gels were released into SF-DMEM with or without salmeterol and/or cilomilast as indicated. Vertical axis: gel size on day 2 (%); horizontal axis: reagents in the medium in which gels were floated. Open bars: cells were cultured in SF-DMEM for 24 hrs prior to being cast into collagen gels; hatched bars: cells were pretreated with 10^−6^ M indomethacin for 24 hrs prior to being cast into collagen gels. *P < 0.05; **P < 0.01.

Since human lung fibroblasts release PGE_2_ and endogenous PGE_2_ may modulate the effect of *β*-agonists by modulating the cAMP signaling pathway, HFL-1 cells were pretreated with indomethacin before being cast into the collagen gels. After the pretreatment with indomethacin (10^−6^ M) for 24 hours, the inhibitory effect of salmeterol on fibroblast-mediated collagen gel contraction was further significantly potentiated (*P* < 0.05, Figure 3(a)). By contrast, there was a slight reduction of contraction in the presence of cilomilast that did not reach statistical significance (Figure 3(b)). In addition, indomethacin pretreatment augmented the inhibitory effect of salmeterol and cilomilast added together (*P* < 0.01, Figure 3(c)). 

**Figure 4:**
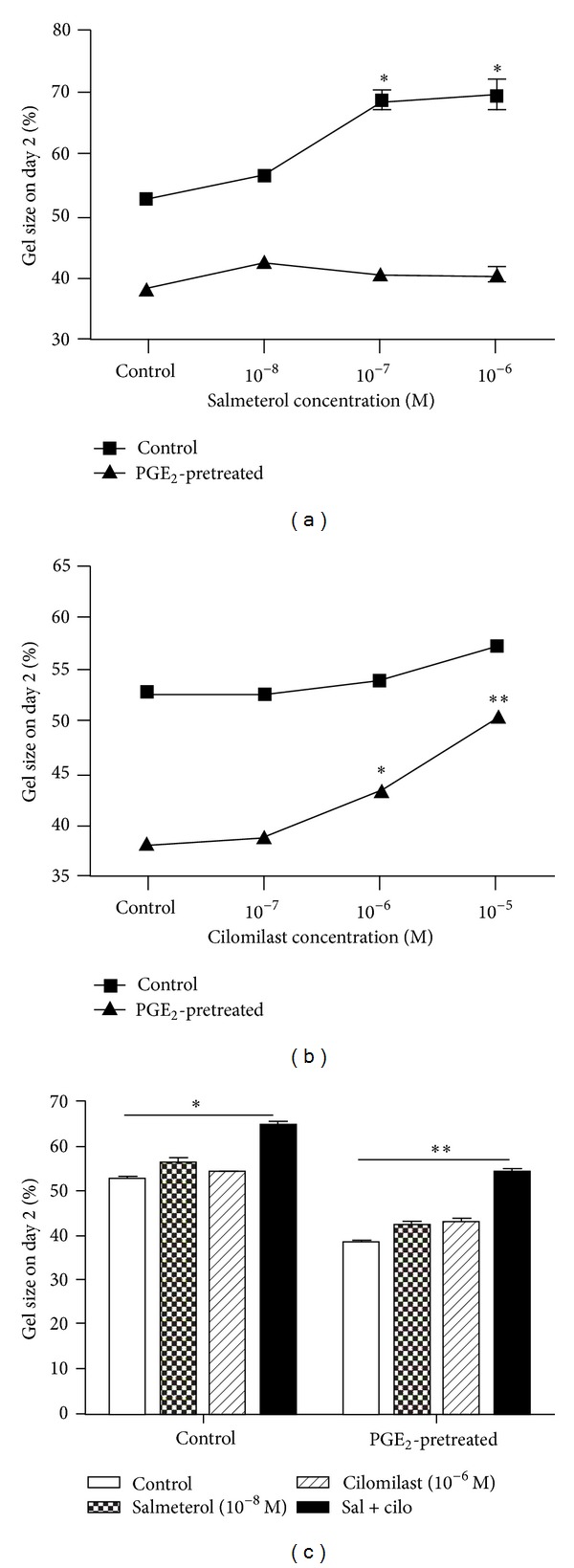
Exogenous PGE_2_ pretreatment potentiates cilomilast but not salmeterol. Confluent HFL-1 cells were pretreated with SF-DMEM with or without 10^−9^ M PGE_2_ for 24 hrs. The next day, cells were cast into collagen gels as described in Section 2. (a) PGE_2_ pretreatment abolished the inhibitory effect of salmeterol on collagen gel contraction. (b) PGE_2_ pretreatment potentiated the effect of cilomilast in inhibiting collagen gel contraction. (c) Effect of PGE_2_ pretreatment on the synergistic effect of salmeterol and cilomilast.

Since the indomethacin pretreatment suggested that endogenous PGE_2_ could attenuate the effect of salmeterol and salmeterol plus cilomilast, we next assessed the effect of exogenous PGE_2_ in modulating salmeterol and cilomilast inhibition of fibroblast-mediated collagen gel contraction. To accomplish this, HFL-1 cells were pretreated with PGE_2_ (10^−9^ M) for 24 hrs prior to being cast into collagen gels and exposed to salmeterol and/or cilomilast. PGE_2_-pretreated cells contracted gels more than the control did cells (Figure 4(a)). By contrast to indomethacin pretreatment, which potentiated salmeterol inhibition of collagen gel contraction, PGE_2_ pretreatment of lung fibroblasts resulted in loss of salmeterol inhibition of fibroblast-mediated collagen gel contraction (Figure 4(a)). By contrast, PGE_2_-pretreatment potentiated the effect of cilomilast when added alone, and after PGE_2_-pretreatment, the inhibition of fibroblast-mediated collagen gel contraction by cilomilast was statistically significant (*P* < 0.05, Figure 4(b)). In cells preexposed to PGE_2_, salmeterol plus cilomilast also significantly inhibited collagen gel contraction (*P* < 0.01, Figure 4(c)).

**Figure 5:**
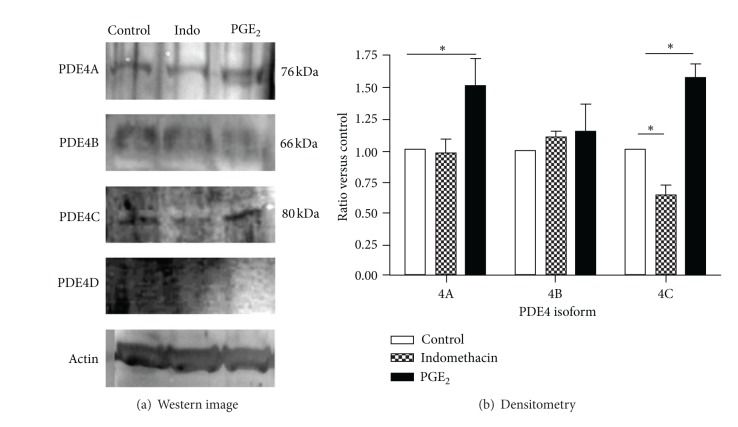
Effect of PGE_2_ and indomethacin on PDE4 expression. Confluent HFL-1 cells were treated with SF-DMEM with or without 10^−9^ M PGE_2_ or 10^−6^ M indomethacin for 24 hrs. Whole cell lysates were immunoblotted for PDE4 isoforms and *β*-actin as loading control. Images were visualized with ECL plus (a), and the bands from 3 separate experiments were quantified (b). *P < 0.05.

To investigate the mechanism by which endogenous and exogenous PGE_2_ modulate long-acting *β*-agonist (salmeterol) and PDE4 inhibitor (cilomilast) inhibition of fibroblast-mediated collagen gel contraction, HFL-1 cells were pretreated with 10^−6^ M indomethacin or 10^−9^ M PGE_2_ for 24 hrs. Whole cell lysate proteins were then extracted and evaluated by immunoblotting for PDE4 isoforms (PDE4A, PDE4B, PDE4C, and PDE4D) with *β*-actin utilized as a loading control. As shown in Figure 5, PDE4A, -4B, and -4C were expressed by HFL-1 cells, while the PDE4D isoform was not expressed (Figure 5(a)). Furthermore, PGE_2_ significantly stimulated PDE4A and -4C (*P* < 0.05) expression, while PDE4B was not affected (Figure 5(b)). Indomethacin, by contrast, inhibited PDE4C expression (*P* < 0.05), but it did not affect PDE4A or -4B. 

## 4. Discussion

Fibroblast-mediated collagen gel contraction is widely used as an *in vitro* model of tissue remodeling. In the current study, using this *in vitro* model of tissue remodeling, we have demonstrated a synergistic effect of the long-acting *β*-agonist salmeterol and the PDE4 inhibitor cilomilast in inhibiting fibroblast-mediated collagen gel contraction. In addition, we have demonstrated that this interaction is modulated by both endogenous and exogenous PGE_2_. Finally, we have demonstrated that PGE_2_ modulates the expression of several PDE4 isoforms, providing a mechanism for regulating the synergistic interaction of salmeterol and cilomilast. 

Consistent with a previous report [7], the long-acting *β*-agonist, salmeterol, inhibited fibroblast-mediated collagen gel contraction at concentrations of 10^−7^ and 10^−6^ M. The PDE4 inhibitor, cilomilast alone, did not affect fibroblast-mediated collagen gel contraction at concentrations of 10^−7^, 10^−6^, and 10^−5^ M. However, when added together, 10^−8^ M salmeterol and cilomilast (10^−7^, 10^−6^, and 10^−5^ M) significantly inhibited collagen gel contraction. Since neither agent alone resulted in inhibition, this demonstrates synergy between these agents. This is consistent with the known role of these agents in modulating cAMP. Salmeterol increases cAMP, and PDE4 degrades cAMP to AMP [19–21]. Cilomilast, by blocking cAMP degradation, could permit accumulation of cAMP concentrations sufficient to inhibit gel contraction in response to concentrations of salmeterol that could not achieve such concentrations alone. Consistent with this, 10^−6^ M cilomilast also potentiated the actions of higher concentrations of salmeterol (10^−8^ and 10^−7^ M) in inhibiting collagen gel contraction.

Several studies have demonstrated that agents that increase cAMP can induce PDE activity [14, 22–28]. Consistent with these studies, pretreatment of the fibroblasts with 10^−6^ M indomethacin for 24 hrs prior to being cast into collagen gels resulted in further inhibition of collagen gel contraction by salmeterol added alone, consistent loss of PDE activity. In contrast, pretreatment with indomethacin did not affect contraction in the presence of cilomilast. 

This contrasts with the effect of pretreatment of the fibroblasts with 10^−9^ M PGE_2_. Interestingly, pretreatment with PGE_2_ resulted in augmentation of contraction. The mechanism of this effect of PGE_2_ pretreatment is not defined, but it resembles the results of Michalski who observed augmentation of chemotaxis following PGE_2_ pretreatment [15]. PGE_2_ pretreatment also changed the response of the fibroblasts to salmeterol and cilomilast added alone. By contrast to control cells, following PGE_2_ pretreatment, cilomilast added alone significantly inhibited gel contraction while salmeterol was without significant effect. This is consistent with the previously observed increase in PDE activity observed in a variety of cell types in response to increases in cAMP [14, 22–28]. Also consistent with such a mechanism, we observed that PDE4C was significantly decreased when the cells were pretreated with indomethacin, while, by contrast, PDE4A and PDE4C were significantly increased when the cells were pretreated with PGE_2_.

Fibroblasts play an important role in tissue remodeling [2]. Long-acting *β*-agonists are widely used in treating COPD for their bronchodilator action [29]. Recently, a PDE4 inhibitor has been approved for use in selected COPD patients to reduce exacerbation risk [30]. Both *β*-agonists and PDE4 inhibitors have the potential to modulate tissue remodeling. Long-acting *β*-agonists have the potential to modulate fibroblast tissue remodeling through inhibiting fibroblast proliferation, differentiation, and collagen production through mechanisms that depend on increasing intracellular cAMP [31]. Phosphodiesterases (PDEs), especially PDE4, may also modulate fibroblast-mediated tissue remodeling by increasing intracellular cAMP levels. In this regard, we have previously reported that *β*-agonists and PDE4 inhibitors could inhibit human lung fibroblast-mediated collagen gel contraction, a widely used model of the tissue contraction that characterizes tissue remodeling [7, 17]. Synergy between a *β*-agonist and a PDE4 inhibitor on fibroblast-mediated collagen gel contraction, however, has not been previously demonstrated. The current study demonstrates such synergy. 

 The present study provides an explanation for differences among prior studies. We have previously reported that cilomilast alone at the concentrations of 10^−7^ ~ 10^−5^ M inhibited fibroblast migration and collagen gel contraction [17], while the same concentrations of cilomilast did not alter fibroblast-mediated collagen gel contraction in the current study. These differing results are likely due to different experimental designs. In the previous report on cilomilast modulation of fibroblast-mediated collagen gel contraction [17], 0.5% FCS was added to the medium in which gels were floated. In the current study, however, there was no serum in the medium in which gels were floated. While the effect of serum is not clear, the current study clearly shows that PGE_2_ can modulate cilomilast responsiveness. Cells pretreated with PGE_2_ were responsive to cilomilast inhibition, which is consistent with PGE_2_ induction of PDE expression. 

Conversely, cells pretreated with PGE_2_ became unresponsive to salmeterol. Consistent with this resulting from the induction of PDE4, the synergistic effect of cilomilast added together with salmeterol was also observed in PGE_2_ pretreated cells. Lung fibroblasts synthesize and release PGE_2_ [9], and the current study also provides evidence that endogenous PGE_2_ can modulate *β*-agonist signaling by affecting PDE4 isoform expression. 

Consistent with previous reports [17], HFL-1 cells expressed predominantly PDE4A, -4B, and -4C but not PDE4D. Interestingly, indomethacin significantly inhibited PDE4C expression, while PGE_2_ significantly stimulated PDE4A and -4C. These findings indicate that PGE_2_, by augmenting PDE4 expression, may accelerate cAMP degradation. Consistent with our findings, it has also been reported that PGE_2_ upregulates PDE4 activity through induction of enzyme synthesis in the myometrium, and by this mechanism PGE_2_ lessens the responsiveness of myometrium to *β*-mimetic activation [16, 22].

Taken together, these results suggest that PDE4 is upregulated by both endogenous and exogenous PGE_2_. In the presence of low levels of PDE4, *β*-agonist (but not PDE inhibitor) can inhibit collagen gel contraction. When PDE4 levels are augmented, however, *β*-agonist becomes less effective since PDE4 results in degradation of any cAMP generated. This loss of responsiveness to *β*-agonist may be particularly important in an inflammatory milieu such as that which exists in COPD where PGE levels are high [32–34]. When added together, there is synergy between *β*-agonist and PDE inhibitor. This suggests that the synergy between PDE4 inhibition and *β*-agonists may be particularly important in COPD where high levels of PGE are present. 

In summary, the current paper demonstrates synergy between a long-acting *β*-agonist (salmeterol) and a PDE4 inhibitor (cilomilast) in their ability to inhibit fibroblast-mediated collagen gel contraction, a widely used model of tissue remodeling. Through such an action, these agents, which are currently in use to treat COPD, could also alter the tissue remodeling present in COPD. In addition, the current study demonstrates that PDE4 expression in lung fibroblasts is induced by PGE_2_ suggesting that this synergy may be particularly important in inflammatory diseases such as COPD where PGE levels are high.
